# Addressing the Key Challenges of Decentralized Clinical Trials in Europe: Multistakeholder Perspective Delphi Study

**DOI:** 10.2196/80625

**Published:** 2026-07-09

**Authors:** Carlos Murciano-Gamborino, Lina Pérez-Breva, Amos J de Jong, Martin Boeckhout, Ghislaine Jose Madeleine Wilhelmien van Thiel, Kate Huntley, Hamidou Traore, Helga Gardarsdottir, Jaime Fons-Martinez

**Affiliations:** 1 Vaccines Research Area, Foundation for the Promotion of Health and Biomedical Research in the Valencian Region (FISABIO-Public Health), Avenida Cataluña, 21, Valencia, 46020, Spain, 34 961925948; 2 Biomedical Research Networking Center for Epidemiology and Public Health (CIBERESP), ISCIII, Madrid, Spain; 3 Julius Center for Health Sciences and Primary Care, University Medical Center Utrecht, Utrecht, The Netherlands; 4 Rotterdam University of Applied Sciences, Rotterdam, The Netherlands; 5 Pfizer R&D UK Limited, Walton-on-the-Hill, United Kingdom; 6 UCB Biopharma, Brussels, Belgium; 7 Division of Pharmacoepidemiology and Clinical Pharmacology, Utrecht Institute for Pharmaceutical Sciences, Utrecht University, Utrecht, The Netherlands; 8 Department of Clinical Pharmacy, Division Laboratory and Pharmacy, University Medical Center Utrecht, Utrecht, The Netherlands; 9 Faculty of Pharmaceutical Sciences, University of Iceland, Reykjavik, Iceland

**Keywords:** decentralized clinical trials, Delphi method, clinical research, digital health, stakeholder engagement, patient-centered trials

## Abstract

**Background:**

Decentralized clinical trials (DCTs) represent an emerging model in clinical research, accelerated by the restrictions imposed during the COVID-19 pandemic. By leveraging digital technologies and local health care resources, DCTs aim to increase accessibility and reduce participant burden compared to traditional site-based models, which often face recruitment failures and high attrition rates. While various regulatory initiatives in Europe, such as the Accelerating Clinical Trials in the European Union program and the European Medicines Regulatory Network recommendation paper (updated in October 2025), have sought to facilitate their implementation, the widespread adoption of DCTs remains limited due to significant operational, regulatory, and technological challenges, including platform fragmentation and gaps in digital literacy.

**Objective:**

This study aimed to identify and prioritize actionable solutions to the main challenges of DCT implementation in Europe from a multistakeholder perspective, gathering insights to address specific ethical, legal, and operational barriers.

**Methods:**

Building on a preceding strengths, weaknesses, opportunities, and threats analysis, a 2-round Delphi study was conducted, involving 26 experts in clinical trials, ethics, law, regulation, and patient engagement between March and May 2023. In the first round, 309 open-ended responses were collected via REDCap (Research Electronic Data Capture; Vanderbilt University) surveys and underwent systematic inductive content analysis using ATLAS.ti (ATLAS.ti Scientific Software Development GmbH) with independent double coding. This process resulted in 244 unique proposals that were categorized according to 6 key challenges. In the second round, 39 synthesized proposals were evaluated using a 4-point Likert scale. Consensus was defined as ≥80% agreement on the appropriateness of each proposal.

**Results:**

High levels of consensus were achieved, with 32 out of the 39 proposals reaching the threshold and 14 achieving 100% unanimity. Overall, 82% of the proposals were rated as “appropriate” or “very appropriate.” Key recommendations included providing support and training for health care professionals, enhancing investigational medicinal product and biological sample logistics through validated technologies, improving collaboration with local health care providers, fostering regulatory harmonization while respecting national specificities, strengthening capacity-building initiatives, and promoting accessible, user-friendly digital tools supported by hybrid trial models. Conversely, proposals such as peer-to-peer participant support and the centralization of ethics reviews at the European Union level failed to reach consensus.

**Conclusions:**

The study offers a prioritized compilation of expert-driven recommendations for overcoming current barriers to DCT implementation in Europe. The adoption of these recommendations could support the development of more inclusive, efficient, and sustainable decentralized research frameworks across diverse health care systems.

## Introduction

### Background

Decentralized clinical trials (DCTs) have emerged as a transformative model in clinical research, accelerated by the restrictions imposed during the COVID-19 pandemic. Unlike traditional site-based designs, DCTs allow participants to undertake study procedures from their homes or community settings by leveraging digital technologies and local health care resources. This shift is primarily driven by the need to overcome the persistent challenges of the conventional trial model, where only half of clinical trials reach their recruitment goal [1,2], often leading to premature discontinuation [2]. From the participant’s perspective, barriers such as time commitment, difficulty traveling to trial sites, and logistical burdens, such as arranging time off work, can deter enrollment [2]. DCTs aim to address these issues by allowing participants to complete activities closer to, or within, their homes [2], promising improvements in trial access, recruitment speed, retention, and population representativeness [1].

In Europe, interest in DCTs has increased substantially. The European Commission and the European Medicines Agency have launched several initiatives to facilitate their adoption, including the Accelerating Clinical Trials in the European Union program [3] and the Recommendation Paper on Decentralized Elements in Clinical Trials issued by the European Medicines Regulatory Network (EMRN) [4]. These initiatives aim to modernize regulatory pathways and provide practical guidance for integrating decentralized elements while safeguarding participant accessibility and compliance. Furthermore, the International Council for Harmonisation of Technical Requirements for Pharmaceuticals for Human Use (ICH) Good Clinical Practice E6(R3) revision [5] supports this model through a proportionate, risk-based approach.

Despite this momentum, the widespread adoption of DCTs remains limited due to significant challenges. Technological limitations—including fragmentation of platforms, lack of interoperability, and limited data standardization—constrain the development of unified digital infrastructures [6,7]. From a methodological perspective, the increasing use of remote data capture requires careful statistical planning, particularly regarding adherence to the ICH estimand framework in late-phase trials [7]. Regulatory agencies such as the Food and Drug Administration have highlighted the need for rigorous evaluation to ensure data quality and participant safety [7].

Operational and ethical considerations also play a crucial role. Although DCTs are expected to enhance patient centricity, recent evidence suggests that remote procedures may introduce new burdens for participants [8] and expand the responsibilities of clinical research staff [9], who increasingly provide technical and organizational support. Ethical concerns related to justice and inclusion are equally salient: digital divides and variability in digital literacy may exacerbate self-selection biases, limiting equitable participation in decentralized studies [10].

Empirical evidence from recent trials, such as the European Trials@Home RADIAL (Remote And Decentralized Innovative Approach to clinical triaLs) proof-of-concept trial [1], a multiarm study conducted in 6 European countries, has empirically validated the persistence and significance of several operational challenges that had been theoretically identified in the strengths, weaknesses, opportunities, and threats (SWOT) analysis that served as the starting point for this study [11] (refer to the *Methods* section).

For example, the implementation of remote electronic consent in the RADIALtrial highlighted obstacles related to technological barriers and regulatory variation [2]. The process proved resource-intensive and was complicated by country-specific consent form layouts and differing regulatory requirements. Furthermore, reliance on electronic identification resulted in some participants needing to revert to remote paper-based consent because of issues with electronic identification or not owning a suitable identification document (eg, lacking a machine-readable zone in the United Kingdom). This specific issue demonstrates the threat of excluding otherwise eligible participants from remote studies if stringent identification protocols are mandated.

Regarding recruitment, online methods (such as search engine advertising and social media) generated the highest awareness, but were significantly less effective at converting this interest into actual trial enrollment compared to outreach through research databases. The challenge of balancing robust prescreening to minimize site burden while maintaining a simple and accessible eligibility assessment for potential participants was evident in the prescreening stage, where a major drop-off occurred: 69% of individuals who started the questionnaire exited at the initial step requiring consent for health data processing. This high attrition rate at the first digital hurdle illustrates the complexity and barriers inherent in the use of digital technologies (Challenge 6).

Although these challenges have been described in various contexts, their precise nature, prioritization, and implications for European implementation remain insufficiently explored. To address this gap, this study applies a Delphi methodology to identify and prioritize actionable solutions from a multistakeholder perspective, including experts in ethics, law, regulation, clinical operations, and patient engagement.

### Study Objectives

This paper explores actionable solutions to these challenges through a classic Delphi methodological approach [12] by gathering insights from experts in ethics, legal frameworks, regulations, clinical operations, and patient advocacy. The research aims to identify and prioritize actionable solutions for advancing DCTs in Europe. Thus, the Delphi method is suitable for answering the research question because it systematically gathers the judgments of different expert groups and can identify agreement and disagreement.

## Methods

### Study Design

**Figure 1.**
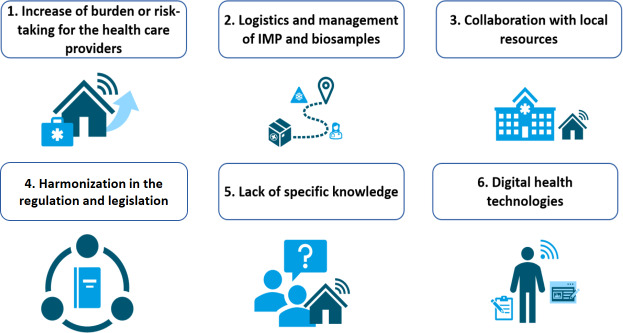
Main areas with challenges for decentralized clinical trials. IMP: investigational medicinal product.

This study focuses on identifying and prioritizing strategies to address the main challenges associated with DCTs through a Delphi approach. Adopting an interpretivist and constructivist epistemological stance, this study used the Delphi method as a consensus-building qualitative approach. This framework acknowledges that actionable solutions for DCT implementation are socially constructed through the subjective expertise of diverse stakeholders, which the researchers systematically interpret and synthesize into a prioritized compilation of recommendations. A previous analysis conducted within the Trials@Home project used a SWOT framework to evaluate key activities in which DCTs differ from conventional trials [11]. The findings from that analysis identified 6 major areas of challenges that need to be addressed by DCTs (Figure 1). Building on these results, the Delphi study was specifically designed to generate and prioritize practical solutions to these challenges.

While the detailed study methodology is comprehensively described in this *Methods* section, a formal, publicly available protocol for this specific Delphi study was not externally preregistered. This decision reflects the study’s primary function as a targeted, consensus-generating activity derived from the Trials@Home project’s preceding analysis (SWOT framework), rather than an independent clinical trial requiring external registration. No external advice was used for the methodology.

### Study Population

Following the criteria outlined by Yáñez Gallardo et al [13], experts were defined as individuals capable of making valuable contributions due to their up-to-date, practice-based knowledge and experience. Accordingly, the expert panel was composed of professionals selected based on their knowledge or relevant experience with clinical trials. Expert profiles included patient representatives, researchers, ethicists, sponsors, regulatory experts, and legal experts. A combined recruitment strategy was used, which included direct email contact with experts identified in the literature or as members of specialized groups (eg, European Federation of Pharmaceutical Industries and Associations DCT subteam), direct contacts through the Trials@Home network, and snowball sampling methods [14].

Although the expert panel was carefully curated to be multidisciplinary (including researchers, ethicists, regulators, legal experts, and patient representatives), it is acknowledged that the composition did not guarantee comprehensive representation of local health care professionals (HCPs), such as community pharmacists or general practitioners (GPs), whose collaboration is fundamental for the effective implementation of DCTs in decentralized settings outside traditional clinical sites. This omission may have inherently skewed the prioritized solutions toward high-level institutional support and harmonization strategies, potentially understating the operational burdens and “last mile” logistical challenges faced by primary care providers.

### Development of the Delphi

The Delphi study was conducted by an interdisciplinary team with representatives from medicine, pharmacy, sociology, political science, and law between March and May 2023. The research team included 2 sociologists with extensive experience in qualitative methods and 4 PhD holders, 3 of whom possessed specific expertise in qualitative research methodologies. The study consisted of 2 consecutive rounds, as described in Textbox 1.

Textbox 1. **Delphi study rounds and procedures.**

**Round 1: generation of proposals**:

- Objective: collect expert-generated proposals addressing the 6 identified challenges.
- Method: open-ended survey administered via REDCap (Research Electronic Data Capture; Vanderbilt University). Each challenge was presented with contextual explanations, followed by open questions requesting specific solutions.
- Analysis: responses were coded and consolidated using content analysis in ATLAS.ti (ATLAS.ti Scientific Software Development GmbH) to remove duplicates and group proposals into thematic categories.

**Round 2: prioritization of proposals**:

- Objective: evaluate and prioritize the proposals obtained in round 1.
- Method: a structured survey in REDCap (Research Electronic Data Capture; Vanderbilt University) presenting grouped solutions and representative examples. Participants rated each proposal on a 4-point Likert scale (1=“not at all appropriate” to 4=“totally appropriate”). In round 2, experts were provided with a list of 39 synthesized proposals, grouped under 6 main challenges (Figure 1), derived from the 244 open responses in round 1. This feedback formed the basis for their evaluation and prioritization in round 2.
- Consensus criteria: consensus was reached when ≥80% of panelists rated a proposal as “appropriate” (3-4) or “not appropriate” (1-2), and this agreement threshold was achieved for ≥80% of all evaluated proposals.

### Data Collection

Data collection was conducted through digital surveys distributed via email in March (invitation to participate and first round of the Delphi study) and May (second round of the study) 2023. The study was planned to run for at least 2 rounds, with the aim of achieving a consensus of at least 80% on all items. This objective was achieved within the minimum number of rounds planned. To ensure a robust sample, the initial response period of 2 weeks was extended to 4 weeks to maximize participation.

### Data Analysis

The data analysis process consisted of two main stages:

1. Qualitative analysis: responses from round 1 were analyzed using a systematic inductive content analysis approach supported by ATLAS.ti to identify common themes and consolidate similar proposals into categories. An open coding process was used, whereby categories and codes emerged organically from the data. Two researchers independently performed the coding, resolving discrepancies by consensus, with the support of a third researcher. Through a constant comparison method, proposals that shared similar objectives or semantic meanings were iteratively grouped and consolidated. This bottom-up approach allowed the research team to refine the list of solutions dynamically as the analysis progressed.
2. Quantitative evaluation: in round 2, proposals were assessed based on their appropriateness using the Bloom cut-off point (cited in Feleke et al [15]):
  - Very appropriate: ≥80% of the maximum score (mean ≥3.40).
  - Fairly appropriate: 65%‐79% of the maximum score (mean 2.95-3.39).
  - Low appropriateness: 50%‐64% of the maximum score (mean 2.5-2.94).
  - Not appropriate: <50% of the maximum score (mean <2.5).

Consensus was considered to be achieved based on scoring concentration (≥80% of panelists) within these categories.

### Ethical Considerations

This study was conducted in accordance with the principles of the Declaration of Helsinki. All participants provided their informed consent before contributing to the Delphi study.

This research did not involve patients and was limited to gathering the professional opinions of expert panelists regarding DCTs. Although participants were identifiable to the research team to confirm consent and track contributions, all responses were pseudonymized for analysis and dissemination, and no sensitive personal data were collected.

As such, the study was not considered to fall within the scope of research requiring ethical review under Spanish law. In Spain, where the research was led, only studies involving biomedical research with human participants or the use of personal data of a sensitive or clinical nature are subject to mandatory ethical review under Law 14/2007 on Biomedical Research. Furthermore, under the European Union General Data Protection Regulation 2016/679 and Organic Law 3/2018, this study posed no risk to the physical or psychological integrity of participants and did not involve any form of clinical intervention or experimentation.

Given that the study aimed to collect expert perspectives for exploratory purposes, did not seek to generate generalizable clinical knowledge, and applied appropriate data protection and ethical safeguards, it was considered exempt from ethical review under the applicable legal and ethical frameworks.

To ensure transparency and adherence to ethical principles in the participation of experts in the Delphi study on DCTs, a carefully structured and personalized recruitment and informed consent process was designed.

The initial contact with potential participants was established via an email sent on behalf of the European project Trials@Home. In this message, the overall aim of the project was introduced. The email clearly and concisely explained the purposes of the Delphi study, detailing how the participants’ expertise and knowledge would be fundamental in identifying solutions to the main ethical, legal, regulatory, and operational challenges faced by DCTs in Europe.

To facilitate informed decision-making, an explanatory leaflet was attached to the email, providing expanded information about the context and methodology of the study. This document describes the prior SWOT analysis conducted to identify the key challenges of DCTs, as well as the process of consulting expert panels. It was explained that, based on this work, 6 main challenges had been defined as the focus of the Delphi. The leaflet also outlined the operation of the Delphi study itself.

The consent process was integrated into several stages. Both the email and the leaflet included a link enabling experts to confirm or decline their participation. Upon acceptance, participants were granted direct access to the first round of the survey. Furthermore, before commencing the questionnaire, the platform displayed an introductory text reiterating the objectives of the study, the structure of the rounds, the estimated time commitment, and assurances of anonymity and confidentiality. Explicit informed consent was requested before proceeding, and it was clarified that participation was entirely voluntary and that personal data would be treated confidentially. The original data would be stored on the Foundation for the Promotion of Health and Biomedical Research in the Valencian Region servers, accessible only to the research team, and the results could be shared with participants after analysis, should they so wish.

## Results

### Overview

A total of 108 emails were sent, of which 36 responses were obtained. Out of these 36 emails, 10 emails declined the invitation; however, specific reasons for nonparticipation were not systematically collected, which is a limitation in assessing potential self-selection bias. The 26 respondents consisted of (nonexclusive categories) researchers (n=13), trial sponsors (n=9), ethicists (n=8), regulators and legal experts (n=5), and patients’ representatives (n=3). Of the 26 panelists, 23 stated that they had experience with DCTs. Regarding demographics, the respondents worked across a diverse set of countries, most frequently Spain (n=5), the United States (n=4), and the Netherlands (n=4), followed by Switzerland (n=2), Italy (n=2), and individual participants from Germany, Portugal, Sweden, Austria, Finland, Denmark, France, and the United Kingdom. In terms of gender distribution, 17 panel members identified as women and 9 as men.

No differentiated subgroup analysis or weighting of responses was performed for any stakeholder, country, or gender category, as the primary objective of this study was to identify a unified multistakeholder consensus.

**Figure 2.**
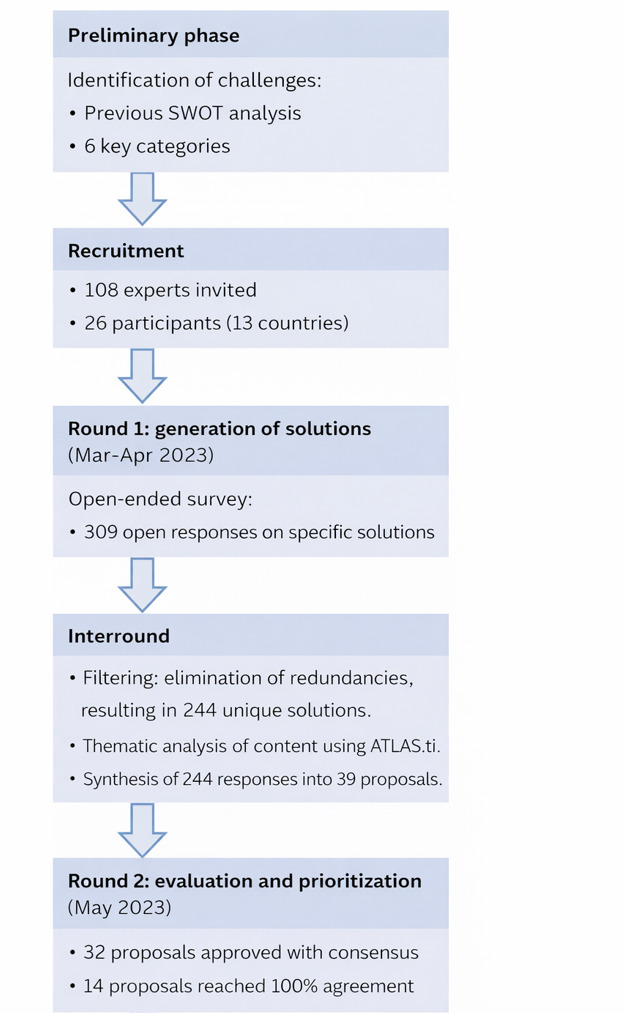
Flowchart illustrating the Delphi study. SWOT: strengths, weaknesses, opportunities, and threats.

Following the DELPHISTAR recommendations [16], a summary of the Delphi study is illustrated in Figure 2 as a flowchart.

### Generating Proposal (Round 1)

From the 26 panelists, a total of 309 open-ended responses were collected as potential solutions to the identified challenges. Following the data cleaning process, 65 entries were excluded because they did not contain concrete proposals or were redundant within the same challenge. The remaining 244 unique solutions underwent a qualitative content analysis using ATLAS.ti software to identify recurring themes and consolidate the data. This process resulted in 39 synthesized proposals across the 6 key challenges. Notably, the qualitative mapping revealed the multidimensional nature of the experts’ insights; while 244 solutions were unique, the analysis identified that 13 of these verbatims had a cross-cutting impact, addressing multiple categories simultaneously (eg, solutions involving both technological automation and risk mitigation). The distribution of these solutions and their consolidation into proposals are summarized in Table 1, while the detailed prioritized proposals and their corresponding verbatims are provided in Table 2 and Multimedia Appendix 1.

**Table 1.** Categorization process for the proposals associated with each challenge.

Description	Ch^a^1	Ch2	Ch3	Ch4	Ch5	Ch6	Total
Potential solutions to the challenges suggested by the panelists (round 1), n	53	45	34	23	37	52	244
Proposals included per challenge in round 2 for evaluation (after the categorization process), n	9	7	5	6	4	8	39

a Ch: challenge.

**Table 2.** Qualitative synthesis: mapping expert verbatims from round 1 to the 39 consensus proposals.

Synthesized proposal (round 2)	Number of verbatims	Illustrative expert verbatim (round 1)
Ch^a^ 1: increase of burden or risk-taking for the HCPs^b^		
Ch1.1: by developing and improving training and support.	16	“Ensure that DCT^c^ provider provides adequate helpdesk support for sites and patients. Ensure that proper training material is generated.” (Expert 7, round 1) “Formal but short education for involved patients has to be established.” (Expert 18, round 1)
Ch1.2. by tailoring trial set-up to DCT elements.	6	“Procedures should be simplified as much as possible, not collect variables that will later have no clinical interest.” (Expert 17, round 1)
Ch1.3: by improving collaboration and involvement of all parties.	7	“The specific roles and responsibilities of the sponsor, investigator, and any additional parties need to be clearly defined in writing and understood.” (Expert 24, round 1)
Ch1.4: through the development and selection of standardized technology.	11	“Use DCT elements from 1 DCT provider via one DCT platform, to avoid that sites and patients need to handle several login data.”(Expert 7, round 1)
Ch1.5: by ensuring remote follow-up of the safety of participants.	10	“Intensive programming in the data collection system is needed to identify adverse effects....Alerts...would be sent to HCP and coordinators for review.” (Expert 12, round 1)
Ch1.6: through the development of a risk mitigation and management plan.	3	“Create a Risk mitigation and management plan which can be shared with sites to provide guidance on how to proactively manage potential or experienced issues.” (Expert 23, round 1)
Ch1.7: by paying more attention to trial safety conditions.	5	“Provide researchers with a safety kit for home visits.” (Expert 9, round 1) “Liability insurance policies...should include legal coverage for all activities performed at the patient’s home.” (Expert 9, round 1)
Ch1.8: through automation of trial procedures.	4	“Create automatic reminders for the research team when no data or bad quality data is coming in...change the activity of data monitoring to a passive instead of an active task.” (Expert 15, round 1)
Ch1.9: by facilitating peer-to-peer support among participants.	1	“Peer to peer support may reduce fears and by that also potential visits.”(Expert 10, round 1)
Ch 2: logistics and management of IMP^d^ and biosamples		
Ch2.1: use of validated products and services.	4	“Only work with qualified service providers that have the required experience to manage such shipping.” (Expert 23, round 1)
Ch2.2: by facilitating IMP management and temperature control.	7	“Transport the IMP or biosamples in boxes which measure the temperature, light, etc, continuously and also upload this to a server.” (Expert 15, round 1)
Ch2.3: by developing training and providing support to participants.	16	“Simplify study kits for participants to easily collect, store and ship samples.” (Expert 14, round 1) “Include an adequate training for participants.” (Expert 25, round 1)
Ch2.4: by adapting the study protocol to the therapeutic area.	8	“If we need a refrigerated centrifuge immediately after the blood sampling, it could be difficult to perform at home....At the end, we need to check every detail.” (Expert 2, round 1)
Ch2.5: by facilitating BioSample management tracking.	2	“QR^e^ codes to track the sample and a way for all parties to see this information.” (Expert 10, round 1)
Ch2.6: training of professionals for new roles in DCTs.	3	“Train the professionals in charge of obtaining, handling and manipulation of biological samples in good clinical laboratory practice standards and IATA^f^.” (Expert 9, round 1)
Ch2.7: using local pharmacies, pick-up points, and health care centers.	7	“Favor easier setting, whenever possible, i.e., local pharmacy to participant, which mimics regular practice for outpatient´s treatment.” (Expert 16, round 1)
Ch 3: effective collaboration with local resources		
Ch3.1: making local HCPs and patients aware of research importance.	3	“The value of research to the participant and wide society should be reinforced. Many HCPs aren’t looking for financial benefits but are looking for benefits to their patients.” (Expert 23, round 1)
Ch3.2: by providing better training and financial resources.	10	“Local resources should be trained and also financially compensated as well as allow them to participate in publications.” (Expert 17, round 1)
Ch3.3: by reducing administrative burdens.	5	“Automate most processes, e.g. add sensors...which inform the researchers when something’s wrong, so that pharmacies or health centers don’t need to check this.” (Expert 15, round 1)
Ch3.4: by describing clearly the roles and responsibilities of partners.	10	“Clear partnerships rather than central command and control.” (Expert 21, round 1) “Contract between local resources and DCT centres.” (Expert 26, round 1)
Ch3.5: by providing incentives and compensation for involvement.	6	“Promote special research center identification for participating centers, eg, with identification plaques on building facades that will result in a better perception by the public.” (Expert 9, round 1)
Ch 4: lack of harmonization in regulation and legislation		
Ch4.1: by developing guidelines and knowledge sharing.	7	“Propose a harmonised guidance, based on interdisciplinary research and stakeholder involvement.” (Expert 25, round 1)
Ch4.2: stimulating learning and harmonization between EU^g^ states.	7	“A common EU approach sets a standard for everyone else to follow. Early release of a guidance means that others can more efficiently follow.” (Expert 23, round 1)
Ch4.3: through gradual implementation of DCT elements.	4	“The first step is to showcase effective DCT elements in local ECs^e^, and drive local changes first.” (Expert 13, round 1)
Ch4.4: by using advanced and verifiable digital security.	3	“The use of advanced and verifiable digital security is essential to allow for e-signatures and protected data collection.” (Expert 14, round 1)
Ch4.5: through specialization in DCT roles.	1	“Ensure to have a dedicated function in the study team for DCT element implementation.” (Expert 7, round 1)
Ch4.6: by centralizing clinical trial ethics review at the EU level.	1	“One review board for all European countries will save time and money for both the researchers and the review boards.” (Expert 15, round 1)
Ch 5: lack of specific knowledge and experience		
Ch5.1: by promoting harmonization of guidelines and dialogue.	10	“Elaborating guidelines like the DCT EMA^h^ guideline, including the different stakeholders involved.” (Expert 3, round 1)
Ch5.2: through knowledge-sharing, education, and training.	21	“Organise sessions or conferences inviting professionals of different profiles to give their vision and talk about their experience.” (Expert 9, round 1)
Ch5.3: by simplifying and optimizing technology to reduce complexity.	1	“Simplify and optimise technology for all parties to reduce complexity.” (Expert 14, round 1)
Ch5.4: by building expertise and moving toward centralized decisions.	5	“Have experts oversee the team and technicians, and use triage for escalation of issues that require expertise.” (Expert 14, round 1)
Ch 6: barriers due to the use of digital technologies		
Ch6.1: by developing and improving training and support for participants.	11	“Ensure proper helpdesk support (in local language!) and training of patients.” (Expert 7, round 1) “Have video explanations available for participants to refer to.” (Expert 14, round 1)
Ch6.2: by ensuring financial and technological resources available.	16	“We have provided hotspots for individuals who do not have wifi.” (Expert 12, round 1) “Sponsor must provide all the devices to patients. Any expense must be reimbursed.” (Expert 3, round 1)
Ch6.3: by simplifying and adapting technology for participants’ use.	6	“Make sure devices work without internet. For example save data offline and upload once internet connection is established.” (Expert 15, round 1)
Ch6.4: by ensuring discussion in the informed consent process.	6	“Ensure that a face to face conversation (includes just telephone but telehealth is now preferred) occurs prior to signing consents.” (Expert 12, round 1)
Ch6.5: by making on-site and offline alternatives available.	5	“The participant should always have the choice to perform assessments also on-site, depending on the patient’s preference.” (Expert 7, round 1)
Ch6.6: by ensuring the data quality of remote and digital technologies.	6	“Real-time data monitoring so that researcher can intervene in time...automate all processes as much as possible to prevent human errors.” (Expert 15, round 1)
Ch6.7: by centralizing the DCT elements in a single vendor.	1	“Use only 1 DCT vendor for all DCT elements, to avoid patients struggling with several systems and log in data.” (Expert 7, round 1)
Ch6.8: through local resources involvement.	3	“Seek the support of local centres for these procedures.” (Expert 9, round 1)

a Ch: challenge.

b HCP: health care professional.

c DCT: decentralized clinical trial.

d IMP: investigational medicinal product.

e QR: quick response.

f IATA: International Air Transport Association.

g EU: European Union.

h EMA: European Medicines Agency.

### Prioritizing Proposals (Round 2)

Of the 26 panelists who participated in round 1, 18 participated in the second survey (round 2). Their self-reported expertise, for which multiple selections were permitted, was distributed as follows: 7 in ethics, 6 in legal, 6 in regulatory, 16 in clinical trials, and 8 in patient engagement aspects. Regarding demographics, the participants represented a diverse international distribution, most frequently working in Spain (n=5), the United States (n=4), Italy (n=2), and the Netherlands (n=2), with individual respondents from Germany, Portugal, Switzerland, Denmark, and France. In terms of gender, 13 participants identified as women and 5 as men.

The ranking of proposed solutions to the respective challenges is provided in Table 3. Proposals with higher mean scores were prioritized accordingly.

**Table 3.** Proposals to overcome each challenge: mean score and interpretation.

Proposal	Mean score (SD)	Score 1‐2, %	Score 3‐4, %	Interpretation
Ch1.1^a^: by developing and improving training and support.	3.67 (0.49)	0	100	Very appropriate With unanimity
Ch1.2: by tailoring trial set-up to DCT^b^ elements.	3.31 (0.48)	0	100	Fairly appropriate With unanimity
Ch1.3: by improving collaboration and involvement of all parties involved in trial conduct.	3.24 (0.56)	6	94	Fairly appropriate With consensus
Ch1.4: through development and selection of more adequate and standardized technology.	3.22 (0.43)	0	100	Fairly appropriate With unanimity
Ch1.5: by ensuring remote follow-up of the safety of participants.	3.17 (0.62)	11	89	Fairly appropriate With consensus
Ch1.6: through the development of a risk mitigation and management plan by the sponsor.	3.12 (0.70)	18	82	Fairly appropriate With consensus
Ch1.7: by paying more attention to trial safety conditions.	3.11 (0.68)	17	83	Fairly appropriate With consensus
Ch1.8: through automation of trial procedures.	2.88 (0.86)	41	59	Low appropriateness Nonconsensus
Ch1.9: by facilitating peer-to-peer support among participants.	2.19 (0.83)	69	31	Not appropriate Nonconsensus
Ch2.1: use of validated products and services.	3.59 (0.62)	6	94	Very appropriate With consensus
Ch2.2: by facilitating IMP^c^ management and temperature control.	3.39 (0.50)	0	100	Fairly appropriate With unanimity
Ch2.3: by developing training and providing support to participants regarding the use of medication and the collection of biological samples.	3.33 (0.59)	6	94	Fairly appropriate With consensus
Ch2.4: by adapting the study protocol to the therapeutic area, participant characteristics, and study procedures.	3.29 (0.59)	6	94	Fairly appropriate With consensus
Ch2.5: by facilitating biological sample management tracking.	3.29 (0.59)	6	94	Fairly appropriate With consensus
Ch2.6: training of professionals for the new roles and delegated tasks of the DCT.	3.28 (0.46)	0	100	Fairly appropriate With unanimity
Ch2.7: using local pharmacies, pick-up points, laboratories, and health care centers.	3.22 (0.73)	17	83	Fairly appropriate With consensus
Ch3.1: making local HCPs^d^ and patients aware of the importance of research for patients.	3.50 (0.52)	0	100	Very appropriate With unanimity
Ch3.2: by providing better training and financial resources for local HCPs.	3.39 (0.61)	6	94	Fairly appropriate With consensus
Ch3.3: by reducing administrative burdens.	3.39 (0.50)	0	100	Fairly appropriate With consensus
Ch3.4: by describing clearly the roles and responsibilities of local partners.	3.39 (	6	94	Fairly appropriate With consensus
Ch3.5: by providing incentives and compensation for the involvement of local resources in trials.	3.17 (0.79)	22	78	Fairly appropriate Nonconsensus
Ch4.1: by developing guidelines and knowledge sharing among stakeholders in DCTs.	3.71(0.47)	0	100	Very appropriate With unanimity
Ch4.2: stimulating learning and harmonization between EU^e^ member states and internationally.	3.41 (0.51)	0	100	Very appropriate With unanimity
Ch4.3: through gradual implementation of DCT elements incorporating adaptations to the local or national specificities.	3.40 (0.51)	0	100	Very appropriate With unanimity
Ch4.4: by using advanced and verifiable digital security.	3.19 (0.75)	19	81	Fairly appropriate With consensus
Ch4.5: through specialization in DCT roles.	3.06 (0.75)	24	76	Fairly appropriate Nonconsensus
Ch4.6: by centralizing clinical trial ethics review at the EU level.	2.76 (0.75)	41	59	Low appropriateness Nonconsensus
Ch5.1: by promoting harmonization of guidelines at the European level and continuous dialogue with regulatory agencies.	3.71 (0.47)	0	100	Very appropriate With unanimity
Ch5.2: through general knowledge-sharing, education, and training for conducting DCTs.	3.47 (0.72)	12	88	Very appropriate With consensus
Ch5.3: by simplifying and optimizing technology to reduce complexity.	3.33 (0.72)	13	87	Fairly appropriate With consensus
Ch5.4: by building expertise on DCTs and moving toward centralized decision making.	3.18 (0.64)	12	88	Fairly appropriate With consensus
Ch6.1: by developing and improving training and support for participants and caregivers.	3.71 (0.47)	0	100	Very appropriate With unanimity
Ch6.2: by making sure sufficient financial and technological resources are available to participants.	3.65 (0.49)	0	100	Very appropriate With unanimity
Ch6.3: by simplifying and adapting technology for participants’ ease of use.	3.53 (0.52)	0	100	Very appropriate With unanimity
Ch6.4: by ensuring that discussion between the researcher and the potential participant is maintained as part of the informed consent process.	3.41 (0.62)	6	94	Very appropriate With consensus
Ch6.5: by making on-site and offline alternatives to decentralized elements available.	3.35 (0.70)	12	88	Fairly appropriate With consensus
Ch6.6: by ensuring data quality of remote and digital technologies used in DCTs.	3.31 (0.60)	6	94	Fairly appropriate With consensus
Ch6.7: by centralizing the DCT elements used in a single vendor.	2.75 (0.77)	31	69	Low appropriateness Nonconsensus
Ch6.8: through local resources involvement	2.69 (0.87)	44	56	Low appropriateness Nonconsensus

a Ch: challenge.

b DCT: decentralized clinical trial.

c IMP: investigational medicinal product.

d HCP: health care professional.

e EU: European Union.

To contextualize the evaluation of proposals presented above, the six main challenges identified through the Delphi process are outlined below:

- Challenge 1: DCTs may increase the burden or risk-taking for health care providers.
- Challenge 2: preventing challenges with logistics and management of investigational medicinal product (IMP) and biosamples.
- Challenge 3: ensuring effective collaboration with local resources.
- Challenge 4: lack of harmonization in regulation and legislation.
- Challenge 5: lack of specific knowledge and accumulated experience for ethical, legal, and regulatory assessment of DCTs.
- Challenge 6: overcoming barriers due to the use of digital technologies.

Considering the percentage of consensus, most of the proposals generated by the panelists during the first round achieved a consensus level exceeding 80%. Specifically, 32 of the 39 proposals fell into this category, 14 of which obtained 100% consensus (unanimity) by being considered as appropriate or very appropriate by all the experts. This high level of agreement indicates that the proposals were not polarized, thereby eliminating the need for a third round of consultations in this Delphi study.

## Discussion

### Principal Findings

Although DCTs may facilitate patient participation through digital technologies and reduce the need for physical visits to research sites, these trials also present a number of challenges that must be addressed to ensure their successful implementation. In this Delphi study, experts identified and evaluated various proposals to mitigate the challenges associated with DCTs.

### Challenge 1: DCTs May Increase the Burden or Risk-Taking for Health Care Providers

DCTs pose the risk of increasing the workload for health care staff [17,18]. Among the most highly valued proposals to mitigate this issue is the provision of adequate support and training.

On one hand, researchers and staff may need to adapt to new regulations, technologies, platforms, and workflows [18-20]. To address these challenges, tools and processes should be designed to minimize barriers to entry and prioritize intuitive, user-friendly interfaces [21]. Furthermore, the EMRN recommendation paper on decentralized elements in clinical trials [4] clearly states that “the sponsor may provide adequate support to trial participants and/or investigators to facilitate the appropriate conduct of their tasks.” The EMRN recommendation paper also allows flexibility for pragmatic implementation of DCT elements, that is, on a “case-by-case” basis, as support provided may be trial-specific. This recommendation underscores the importance of providing support not only for technological and digital adaptation but also for procedural aspects that enhance the overall experience of (site) staff in DCTs.

In the context of DCTs, the term “risk-taking” for health care providers encompasses a variety of potential risks, including increased responsibilities due to new procedures or technologies that may fall outside their traditional scope of practice; operational risks such as conducting study procedures in participants’ homes or in remote settings, where the environment is less controlled than in clinical sites; technological risks including potential failure of digital platforms or devices, which may disrupt workflows or data collection; and risks to personnel safety and well-being, such as increased stress from remote monitoring, ergonomic issues from working outside the usual clinical environment, or, in rare cases, personal safety concerns when visiting participants’ homes.

Interestingly, while the EMRN recommendations emphasize that DCTs should not increase risk, the literature tends to focus on risks such as data security [22-26], the safety of the IMP, or participant safety [19]. Possible risks for future patients, such as those associated with implementing health interventions based on potentially inaccurate research results, are also discussed in the literature [27]. In our study, 2 recommendations deemed “fairly appropriate” included developing a risk mitigation or management plan by the sponsor and paying greater attention to trial safety conditions. These measures include common risk mitigation procedures, such as involving investigators and research teams early in study design to identify risks from the outset. Risk mitigation plans may also integrate a thorough risk and benefit assessment into protocols for early regulatory acceptance and provide guidance for investigators, potentially as part of study initiation training, to proactively address challenges in DCTs. In some circumstances, the risk may be the same as in an on-site visit, but staff may perceive that performing certain activities outside the environment to which they are used may increase their risk. However, risks related to staff safety or well-being remain scarcely mentioned in the literature, highlighting this as an underexplored area.

On the other hand, one proposal considered “not appropriate” (with nonconsensus) by our panelists for addressing this specific challenge (increased burden) was facilitating peer-to-peer support among participants. While participants often face barriers to accessing DCTs and require training and support for specific tasks, whether digital or otherwise, there is skepticism regarding whether peer-to-peer training can effectively reduce the burden on health care staff in this activity. Some concerns include whether such an approach might inadvertently introduce new challenges [28,29]. Nevertheless, some studies contradict this perspective by demonstrating that peer-to-peer training can yield promising results that benefit both participants and researchers [30,31]. Although the experts in this study did not consider this strategy suitable for overcoming this particular challenge, it could offer other advantages for participants that were not explored in this study [29].

### Challenge 2: Preventing Challenges With Logistics and Management of IMP and Biosamples

The management of IMPs and biological samples is perceived by experts as a critical and sensitive component in DCTs. Key concerns include temperature deviations during transit, traceability risks, last-mile delivery issues, and operational burdens related to the delegation of new roles. These challenges are not exclusive to DCTs and have also been observed in conventional clinical trials; however, their impact may be magnified in decentralized settings due to the complexity and fragmentation of logistics chains.

Ensuring the stability and traceability of IMPs requires both technological innovation and logistical rigor. Among the most highly rated recommendations by the panel was the need to facilitate temperature control throughout the entire distribution process. This is essential, as any deviation from required conditions can lead to the quarantine or loss of IMPs, impacting patient access and incurring additional costs. The implementation of advanced monitoring systems, such as internet of things–enabled cold chain systems using wireless sensors and radio-frequency identification technology, can provide real-time alerts and enable retrospective tracking of environmental conditions. Cil et al [32] demonstrated, in the context of container ports, that these systems significantly reduce temperature deviations in pharmaceutical logistics—a finding that can be extrapolated to DCTs given the similar cold chain requirements.

Notably, while the panel reached consensus on the importance of temperature control and validated delivery services, they did not define universal technical thresholds, such as specific temperature excursion limits or escalation procedures. Consistent with the EMRN recommendation paper [4], the appropriateness of IMP delivery and storage arrangements should be supported by a trial-specific risk assessment taking into account factors such as the IMP’s stability, storage conditions, and logistical robustness, and should be described in the clinical trial protocol or related documentation.

Another recommendation from the experts was to facilitate biological sample management tracking. According to Bamakan et al [33], blockchain-based frameworks prevent unauthorized data modifications and ensure transparency throughout the pharmaceutical supply chain. The potential of these technologies to enhance trust and traceability is considerable, especially in decentralized models where oversight is more distributed. Blockchain can serve as a backbone for reliable data logging, helping ensure that every step in biological sample collection, storage, and transport is securely recorded and auditable.

The use of validated products and services was the highest-ranked proposal made by the experts to overcome these challenges. This recommendation underscores not only the importance of technology but also the value of collaborating with specialized logistics providers as a robust strategy to mitigate operational risks [4,34]. Several companies offer Good Distribution Practice–compliant logistics services tailored to clinical trials [35-38]. These services typically include temperature-controlled transport, real-time monitoring dashboards, contingency planning for temperature excursions, and end-to-end chain-of-custody documentation.

Another potential solution to challenges with IMP logistics in the context of a DCT is the use of local pharmacies, laboratories, and health care centers as IMP pick-up points, as also proposed in the EMRN recommendation paper [4]. While this was rated as moderately appropriate by our expert panel, several barriers were noted. Regulatory restrictions on vendor authorization in some European countries, increased burden on local staff, challenges in supervising external contributors to the DCT process, and labeling requirements represent significant obstacles to implementation. Additionally, while local resources may offer potential solutions, their effective use is hindered by risks associated with additional intermediaries in the supply chain. Each transfer point increases the likelihood of uncontrolled temperature exposure, potentially compromising the IMP’s stability, safety, and efficacy. Complex logistics may heighten the risk of undocumented temperature excursions and traceability errors, particularly in the “last mile,” where temperature monitoring is often inadequate [39]. Variability in surveillance methods among stakeholders further complicates data consolidation, creating uncertainty about the IMP’s viability at administration. While local pick-up points may be viable in some cases, their use must be supported by stringent temperature control and auditing protocols to ensure product integrity and minimize operational risks.

To ensure robust IMP and biological sample management, a coordinated framework is needed that includes not only the use of external technologies and services but also the development of training programs for participants regarding medication use and biological sample collection. Additionally, professionals should be trained for new roles and delegated tasks specific to DCTs.

### Challenge 3: Ensuring Effective Collaboration With Local Resources

Collaboration with local health care providers—such as GPs, home health care services, community pharmacists, and local hospitals—can facilitate the implementation of DCTs that might otherwise be unfeasible. This is particularly true for procedures that cannot be conducted at home or are unsuitable for specific therapeutic areas or patient populations [40,41].

The effective integration of these local resources hinges not only on their involvement but also on the adoption of digital technologies and the provision of adequate training. These elements are essential to empower local stakeholders to carry out their roles efficiently within DCT frameworks. Reflecting broader trends in stakeholder engagement, the panelists identified raising awareness as one of the most highly valued strategies—for example, through digital campaigns or community volunteer programs (eg, volunteers based in community pharmacists) [42]. However, such initiatives are most impactful when supported by complementary measures, including enhanced funding, incentive structures, and compensation mechanisms, which are seen as vital to fostering sustained and meaningful collaboration [43,44]. Surprisingly, despite the emphasis in the literature on these support structures, our findings revealed a lack of expert agreement on this point: the proposal to provide incentives and compensation for the involvement of local resources (Challenge 3.5) was rated only as “fairly appropriate” and was one of the few items that failed to reach consensus. This result highlights a significant nuance in addressing the barrier of effective local collaboration: while incentives are often proposed to increase interest, qualitative evidence suggests they may prove ineffective if the underlying obstacles are structural rather than motivational [45].

General hospitals and nonacademic health centers often encounter more pronounced challenges compared to university-affiliated institutions. These include workforce limitations, low clinical trial awareness among both patients and clinicians, and logistical obstacles such as transportation barriers [46]. These difficulties may be exacerbated in environments lacking experienced DCT professionals or where there is institutional resistance to adopting new technologies [47]. In such cases, collaboration with local contributors to the DCT process underscores the critical need for tailored support, investments in training programs, and financial resources to build and strengthen local capacities—recommendations endorsed by all experts consulted in our study.

Administrative complexity is another significant barrier that can hinder local engagement in DCTs [48]. To address this, panelists recommended reducing administrative burdens, particularly by improving data interoperability—such as through initiatives such as the European Health Data Space [49]—local GPs, pharmacists, and small hospitals often lack the time and resources to handle complex documentation. European Health Data Space can make its participation more feasible. Such measures would help mitigate disparities in professional expertise and role definitions across different countries, ultimately promoting more efficient cross-border collaboration.

Consistent with the EMRN recommendation paper [4], the clear delineation of roles and responsibilities among local partners emerged as a key recommendation. The EMRN advises that when trial-specific tasks are delegated to service providers, their responsibilities should be outlined at a high level within the trial protocol and further detailed in supporting documents. Moreover, a formal agreement must be established between the responsible party (as defined by ICH E6) and the service provider to ensure accountability and compliance.

Although the experts emphasized the importance of training (Challenge 3.2), they did not define minimum qualification thresholds or licensure requirements for personnel. In alignment with the EMRN recommendation paper [4], the sponsor should ensure that any contracted service provider is qualified for the delegated tasks, while the investigator must be informed of their qualifications and retain the right to agree or reject the provider, maintaining ultimate responsibility for participant safety.

### Challenge 4: Lack of Harmonization in the Regulation and Legislation

The regulatory landscape, encompassing legislation and guidance, plays a pivotal role in shaping the implementation of DCTs. However, significant variability across jurisdictions regarding the regulation of core DCT components presents a major challenge, particularly in multicountry trials. In some cases, there is a complete absence of relevant regulation, which affects critical areas such as the distribution, return, and destruction of IMPs [19,20,50,51], the qualifications and scope of responsibilities of HCPs [19,20], and the use of digital applications and technologies [52].

In response, regulatory authorities have begun addressing these discrepancies by issuing tailored guidance for decentralized trials [4]. However, our expert panelists emphasized that addressing regulatory harmonization requires more than nonbinding guidance documents; it also demands proactive knowledge-sharing among all stakeholders involved in DCTs (eg, regulatory authorities, ethics committees, HCPs, academic institutions, sponsors, and so on). This combined approach, harmonized guidance, and collaborative learning, was unanimously considered as a highly appropriate strategy to overcome current regulatory barriers. In practice, this refers to measures such as engaging with DCT vendors early in the planning process to obtain up-to-date regulatory intelligence, adapting trial protocols to the specific requirements of each country, avoiding reliance on static internal regulatory information repositories, and establishing neutral, multistakeholder platforms to foster ongoing dialogue and the co-design of new procedures and frameworks that support the diverse applications of DCTs.

In addition, the panel unanimously valued the gradual implementation of DCT elements incorporating adaptations to local or national specificities as a particularly suitable approach. This involves the incremental integration of decentralized components—such as remote consent, telemedicine, at-home drug delivery, and digital patient monitoring—tailored to the regulatory and operational context of each jurisdiction. Such a pragmatic strategy aligns with the growing trend toward hybrid clinical trial models, enabling sponsors to ensure regulatory compliance and operational feasibility while progressively advancing toward full decentralization [19,53]. The Recommendation Paper on Decentralized Elements in Clinical Trials offers in its appendix a comprehensive “National Provisions Overview” mapping key DCT elements—such as remote consent, home nursing, and direct-to-patient IMP delivery—against specific country-level constraints and requirements [4].

The implementation of advanced and verifiable data security protocols was also identified as a key enabler of DCTs. This aligns with the European Medicines Agency’s Guideline on computerized systems and electronic data in clinical trials [54], which emphasizes the importance of data integrity based on Attributable, Legible, Contemporaneous, Original, Accurate, Complete, Consistent, Enduring, Available principles. Digital tools such as Electronic Clinical Outcome Assessment, electronic case report form, wearable devices, and interactive response technology must undergo robust validation, feature stringent access controls and audit trails, and incorporate comprehensive security mechanisms to prevent unauthorized changes and ensure traceability. Secure data transmission and storage, coupled with routine backup and compliance checks, are critical to ensuring the reliability and credibility of trial data. Regular audits and oversight further reinforce data quality standards across DCT settings.

Ethical oversight presents another layer of complexity. The fragmentation of research ethics committees across different jurisdictions often necessitates multiple approvals for a single decentralized study. While the centralization of ethical review through a unified European research ethics committee could streamline these processes and foster consistent ethical interpretations [55], our panelists did not consider this approach sufficiently appropriate and did not reach a consensus on the matter. Their reservations may reflect a broader inclination to preserve national competencies in ethically and culturally sensitive domains. Current European legislation [56] promotes cooperation and harmonization of certain procedures between Member States, but still recognizes the importance of each country retaining authority over the assessment of ethical and contextual aspects. This hybrid approach, balancing European coordination with respect for national autonomy, may contribute to a preference for ethics committees and experts maintaining competencies at the national level, especially with regard to the interpretation and application of ethical principles influenced by each Member State’s legal and cultural framework.

The proposal to create specialized DCT roles (Challenge 4.5) emerged from initial stakeholder insights regarding increased operational complexity driven by the current variety of technologies and regulations, suggesting a need for dedicated functions to ensure correct implementation. However, when this proposal was evaluated in the second round, it was rated only as “fairly appropriate” and failed to reach the consensus threshold (76%). This lack of agreement may reflect a divergence of opinion on whether such specialization is practically viable at the site level or whether it risks fragmenting the investigator’s ultimate oversight responsibilities as defined by regulatory standards [4].

### Challenge 5: Lack of Specific Knowledge and Accumulated Experience for the Ethical, Legal, and Regulatory Assessment of DCTs

The implementation of DCTs faces challenges due to limited experience and variability in ethical, legal, and regulatory interpretations across European countries. This heterogeneity can lead to inconsistencies in trial approvals, delays in implementation, and difficulties in ensuring compliance with Good Clinical Practice standards. To address these issues, both the literature and our panel of experts emphasize that, while harmonized European Union–level frameworks are essential, their effective implementation requires ongoing dialogue and coordination with national regulatory authorities, as well as mechanisms to facilitate knowledge sharing and alignment of practices across countries [19,57,58].

An essential pillar for ensuring effective oversight and compliance with DCT regulatory requirements is capacity building through knowledge sharing, education, and training for all stakeholders involved [19,55,59]. This strategy was identified as a very appropriate recommendation by our panelists. Strengthening capacity-building initiatives among researchers, ethics committees, and regulatory authorities is crucial for improving the evaluation and implementation of DCTs.

Training programs focused on digital methodologies, remote patient monitoring, and data integrity can help bridge knowledge gaps and foster confidence in decentralized trial approaches. Moreover, harmonized regulatory frameworks not only streamline approval processes but also create a foundation for collaborative learning and best-practice dissemination across Member States. In this context, European initiatives may play a pivotal role in ensuring that stakeholders are well-equipped to navigate the evolving landscape of DCTs.

Thus, a proposal rated as fairly appropriate by the panelists was the simplification and optimization of technology to reduce the complexity of its ethical, regulatory, and legal assessment. This aligns with the existing trend toward simplification of processes following the Keep It Simple, but Smart principle [60] (more commonly cited as Keep It Simple, Stupid [61]) or the demand for increasing transparency, as reflected in the international standard EN 62304, Regulation (EU) 2017/745 of the European Parliament and of the Council of April 5, 2017, on medical devices or Regulation (EU) 2024/1689 of the European Parliament and of the Council of June 13, 2024, laying down harmonized rules on artificial intelligence.

### Challenge 6: Overcoming Barriers Due to the Use of Digital Technologies

DCTs are defined by the relocation of certain trial activities from traditional research sites to participants’ own environments, a shift largely enabled by digital technologies and remote communication tools [62]. However, this transformation is not without its challenges, many of which require targeted strategies to ensure inclusivity, usability, and ethical integrity.

One of the most pressing priorities, according to our panelists, is the development and enhancement of training and support systems for participants and their caregivers. This aligns with current literature highlighting the critical role of both digital and health education in the successful adoption of decentralized trial methods [63-65].

A notable design model in some DCTs is the “bring your own device” approach, where participants use their personal devices to engage with the study. While this model may offer flexibility, it risks introducing participation bias due to unequal access to technology [7]. To mitigate this, experts strongly recommended ensuring adequate financial and technological support for all participants. Importantly, both the bring your own device model and the provision of sponsor-supplied devices raise distinct ethical considerations that must be addressed transparently [66].

Another proposal explored by the panel was the centralization of digital trial components within a single vendor. While this approach received lower appropriateness ratings overall (with nonconsensus), it was deemed more feasible in scenarios where all devices are supplied by the sponsor. This finding is significant as it diverges from the literature, which argues that centralizing technology under one vendor could facilitate smoother integration of data systems and user interfaces across stakeholders—sponsors, research sites, and participants—potentially enhancing the overall efficiency and user experience of the trial [6].

Simplifying and tailoring digital tools to the needs of participants was identified as a highly appropriate recommendation, particularly in light of the principle of justice. Usability improvements—such as intuitive interfaces, familiar device formats, timely feedback, and participant notifications—not only enhance adherence, especially when participants are involved in the design process [67], but also help ensure equitable access. Technologies that feature simple user interfaces, sufficient data storage capacity, and minimal requirements for frequent updates enable individuals with low digital literacy or limited devices (such as basic smartphones) to participate more equally in clinical trials, thereby facilitating a fairer and more inclusive ethical assessment process [6,47].

Equally important is preserving the interpersonal dimension of the informed consent process. Our panelists stressed that even when using electronic consent formats, direct communication between the investigator and the potential participant must be maintained. This view echoes findings from the i-CONSENT project (Improving the guidelines for Informed Consent, including vulnerable populations, under a gender perspective), which distinguishes between the provision of information (digital or otherwise) and the essential human dialogue that underpins truly informed consent [68].

This position is further supported by the EMRN’s recommendation paper on DCTs [4], which states that remote informed consent processes should include audio-visual interaction, secure encrypted channels, and identity verification. Furthermore, when trial populations are particularly vulnerable, when the investigational product has limited safety or efficacy data, or when trial procedures are complex or high-risk, a physical meeting between participant and investigator becomes even more critical and should be strongly considered.

Finally, the importance of providing on-site or offline alternatives to decentralized procedures was also underscored by both the EMRN and our panelists. While the EMRN focuses specifically on in-person options for informed consent, broader literature, such as the work by Kruse et al [63], supports the idea of hybrid models—combining telemedicine and traditional in-person care—as a viable strategy to promote technological accessibility and improve participant retention.

### Study Limitations

First, the Delphi panel, although composed of diverse experts, remains relatively small (n=26 in round 1 and n=18 in round 2) and may not capture the full breadth of perspectives across all European countries or stakeholder groups. Second, participation was voluntary and may reflect selection bias toward individuals already interested in DCTs. Third, the Delphi methodology relies on expert opinion rather than empirical measurement, and consensus does not necessarily equate to correctness or feasibility in real-world settings. Fourth, the study focused on perceived challenges and solutions rather than evaluating their actual implementation or effectiveness. Finally, because the research was conducted within the context of a European consortium, the findings may not fully generalize to non-European environments with different regulatory or health care structures.

### Conclusion

This Delphi study has enabled the identification and prioritization of concrete proposals to address the main challenges associated with DCTs from a multistakeholder perspective. Through a structured methodological approach, a high level of consensus was reached among experts in ethical, legal, regulatory, operational, and patient engagement domains, thereby reinforcing the validity of these findings for the consulted panel. The findings underscore the need to strengthen support for HCPs, optimize the logistical management of IMPs and biological samples, and enhance collaboration with local resources through appropriate incentives and reduced administrative burdens. Advancing regulatory harmonization across Europe—through a combination of shared guidance and respect for national competencies—was also identified as a priority, alongside the promotion of ongoing training to address the lack of accumulated experience. In the technological domain, ensuring accessibility, usability, and adequate support for participants was highlighted as essential, as was maintaining the interpersonal dimension of the informed consent process.

Taken together, the proposals gathered in this study represent a snapshot of the consensus achieved by this multidisciplinary group between March and May 2023, providing a pragmatic foundation for fostering a more equitable, safe, and efficient implementation of DCTs across Europe. While these findings offer valuable guidance, they should be interpreted as preliminary consensus insights that serve as a basis for future research and adaptations as regulations, technologies, and site capabilities continue to evolve.

## Supplementary material

10.2196/80625 Multimedia Appendix 1 Full list of expert verbatim responses from round 1 of the Delphi study, organized by the 6 identified challenges.

## Abbreviations

DCT: decentralized clinical trial
EMRN: European Medicines Regulatory Network
GP: general practitioner
HCP: health care professional
ICH: International Council for Harmonisation of Technical Requirements for Pharmaceuticals for Human Use
IMP: investigational medicinal product
RADIAL: Remote And Decentralized Innovative Approach to clinical triaLs
REDCap: Research Electronic Data Capture
SWOT: strengths, weaknesses, opportunities, and threats

## References

[1] Zuidgeest MGP, Heath M, Lagerwaard B (2025). Bringing trial activities to participants-the Trials@Home RADIAL proof-of-concept trial investigating decentralization of trials. Clin Pharmacol Ther.

[2] Lagerwaard B, Rutgrink L, van Weelij D (2025). Recruiting and consenting decentralized clinical trial participants—learnings from the Trials@Home RADIAL proof‐of‐concept trial. Clin Pharma and Therapeutics.

[3] (2022). Accelerating clinical trials in the EU (ACT EU): delivering an EU clinical trials transformation initiative. https://www.ema.europa.eu/en/documents/regulatory-procedural-guideline/accelerating-clinical-trials-eu-act-eu-delivering-eu-clinical-trials-transformation-initiative_en.pdf.

[4] (2025). Recommendation paper on decentralised elements in clinical trials. https://health.ec.europa.eu/system/files/2023-03/mp_decentralised-elements_clinical-trials_rec_en.pdf.

[5] (2025). E6(R3) Good Clinical Practice (GCP). US Food & Drug Administration.

[6] Copland RR, Hanke S, Rogers A (2024). The digital platform and its emerging role in decentralized clinical trials. J Med Internet Res.

[7] Chen J, Di J, Daizadeh N (2025). Decentralized clinical trials in the era of real‐world evidence: a statistical perspective. Clinical Translational Sci.

[8] Vayena E, Blasimme A, Sugarman J (2023). Decentralised clinical trials: ethical opportunities and challenges. Lancet Digit Health.

[9] Gamble E, Heavin C, Linehan C (2025). Adaptation of clinical research staff to decentralized clinical trials and impacts on the patient-centered experience: qualitative interview study. J Med Internet Res.

[10] van Rijssel TI, van Delden JJM, Lagerwaard B, Zuidgeest MGP, van Thiel G, Trials@Home consortium (2025). Diversity in decentralized clinical trials: prioritizing inclusion of underrepresented groups. BMC Med Ethics.

[11] Murciano-Gamborino C, Pérez-Breva L, de Jong AJ (2026). SWOT analysis of decentralised clinical trials from an ethical, legal, regulatory and operational perspective. BMC Med Ethics.

[12] Hasson F, Keeney S, McKenna H (2025). Revisiting the Delphi technique - research thinking and practice: a discussion paper. Int J Nurs Stud.

[13] Yañez Gallardo R, Cuadra Olmos R (2008). La técnica Delphi y la investigación en los servicios de salud. Cienc Enferm.

[14] Lewis-Beck MS, Bryman A, Liao TF (2004). The SAGE Encyclopedia of Social Science Research Methods.

[15] Feleke BT, Wale MZ, Yirsaw MT (2021). Knowledge, attitude and preventive practice towards COVID-19 and associated factors among outpatient service visitors at Debre Markos compressive specialized hospital, north-west Ethiopia, 2020. PLoS One.

[16] Niederberger M, Schifano J, Deckert S (2024). Delphi studies in social and health sciences—recommendations for an interdisciplinary standardized reporting (DELPHISTAR). Results of a Delphi study. PLoS One.

[17] de Jong AJ, van Rijssel TI, Zuidgeest MGP (2022). Opportunities and challenges for decentralized clinical trials: European regulators’ perspective. Clin Pharmacol Ther.

[18] (2022). Site perspectives on decentralized clinical trials. https://www.medidata.com/wp-content/uploads/2022/10/Site-Perspectives-on-Decentralized-Clinical-Trials-Sep-22.pdf.

[19] Apostolaros M, Babaian D, Corneli A (2020). Legal, regulatory, and practical issues to consider when adopting decentralized clinical trials: recommendations from the clinical trials transformation initiative. Ther Innov Regul Sci.

[20] Hirsch IB, Martinez J, Dorsey ER (2017). Incorporating site-less clinical trials into drug development: a framework for action. Clin Ther.

[21] Leighton M (2022). Overcoming the 7 biggest challenges in launching decentralized trials. Applied Clinical Trials.

[22] Mathieu E, Barratt A, Carter SM, Jamtvedt G (2012). Internet trials: participant experiences and perspectives. BMC Med Res Methodol.

[23] Steinhubl SR, Wolff-Hughes DL, Nilsen W, Iturriaga E, Califf RM (2019). Digital clinical trials: creating a vision for the future. NPJ Digit Med.

[24] Hand S (2018). Virtual clinical trials – can remote trials change the clinical trial landscape?. Xtalks.

[25] Pfaff E, Lee A, Bradford R (2019). Recruiting for a pragmatic trial using the electronic health record and patient portal: successes and lessons learned. J Am Med Inform Assoc.

[26] Nicholas A, Bailey JV, Stevenson F, Murray E (2013). The Sexunzipped trial: young people’s views of participating in an online randomized controlled trial. J Med Internet Res.

[27] Andrews L, Kostelecky K, Spritz S, Franco A (2017). Virtual clinical trials: one step forward, two steps back. J Health Care Law Policy.

[28] Simmons D, Bunn C, Nakwagala F (2015). Challenges in the ethical review of peer support interventions. Ann Fam Med.

[29] Joo JH, Bone L, Forte J, Kirley E, Lynch T, Aboumatar H (2022). The benefits and challenges of established peer support programmes for patients, informal caregivers, and healthcare providers. Fam Pract.

[30] Tang PY, Duni J, Peeples MM (2021). Complementarity of digital health and peer support: “This Is What’s Coming”. Front Clin Diabetes Healthc.

[31] Sheikh SZ, Donovan C, Menezes C (2023). Feasibility and utility of a pilot peer education program to improve patient engagement in lupus clinical trials: implementation and evaluation in a multisite model within a lupus clinical trials network. ACR Open Rheumatol.

[32] Cil AY, Abdurahman D, Cil I (2022). Internet of Things enabled real time cold chain monitoring in a container port. J Shipp Trade.

[33] Bamakan SMH, Moghaddam SG, Manshadi SD (2021). Blockchain-enabled pharmaceutical cold chain: applications, key challenges, and future trends. J Clean Prod.

[34] Redding S, Sweeney M (2016). The growth of direct-to-patient trials. Applied Clinical Trials.

[35] (2022). Why cold chain logistics matters for the healthcare industry. DHL.

[36] Leading pharmaceutical logistics and cold chain solutions for life sciences. Biocair.

[37] Homepage. UPS Healthcare.

[38] Beh C (2021). What does the future of cold chain shipping look like?. FedEx.

[39] Peter M, Yusef N, Sadler-Williams E, Stanbrook R, Carmichael S, Cunnington G (2019). A new approach to temperature monitoring in a changing clinical supply chain environment. Applied Clinical Trials.

[40] Rodrigo-Casares V, Perez-Breva L, Fons-Martinez J Ensayos clínicos descentralizados. https://ancei.es/wp-content/uploads/2022/05/LibroPonencias.pdf.

[41] How decentralized and hybrid trials accelerate research and enable diversity. Applied Clinical Trials.

[42] Dahrouge S, Kaczorowski J, Dolovich L (2018). Long term outcomes of cluster randomized trial to improve cardiovascular health at population level: The Cardiovascular Health Awareness Program (CHAP). PLoS One.

[43] Schell D (2025). Local HCPs in DCTs: big benefits, bigger burdens. Clinical Leader.

[44] Addala A, Hechavarria M, Figg L (2023). Recruiting historically under-represented individuals into Project ECHO Diabetes: using barrier analysis to understand disparities in clinical research in the USA. BMJ Open.

[45] Team V, Bugeja L, Weller CD (2018). Barriers and facilitators to participant recruitment to randomised controlled trials: a qualitative perspective. Int Wound J.

[46] Ebrahimi H, Megally S, Plotkin E (2024). Barriers to clinical trial implementation among community care centers. JAMA Netw Open.

[47] Rogers A, De Paoli G, Subbarayan S (2022). A systematic review of methods used to conduct decentralised clinical trials. Br J Clin Pharmacol.

[48] Easy M (2023). The key to reducing administrative burden for clinical trials. Physicians Practice.

[49] European Commission (2022). Proposal for a regulation of the European Parliament and of the council on the European Health Data Space. COM(2022) 197 final. Eur-Lex.

[50] de Jong AJ, Santa-Ana-Tellez Y, Zuidgeest MGP (2023). Direct-to-participant investigational medicinal product supply in clinical trials in Europe: exploring the experiences of sponsors, site staff and couriers. Br J Clin Pharmacol.

[51] Orri M, Lipset CH, Jacobs BP, Costello AJ, Cummings SR (2014). Web-based trial to evaluate the efficacy and safety of tolterodine ER 4 mg in participants with overactive bladder: REMOTE trial. Contemp Clin Trials.

[52] Vogel MME, Combs SE, Kessel KA (2017). mHealth and application technology supporting clinical trials: today’s limitations and future perspective of smartRCTs. Front Oncol.

[53] Hanley DF, Bernard GR, Wilkins CH (2023). Decentralized clinical trials in the trial innovation network: value, strategies, and lessons learned. J Clin Trans Sci.

[54] (2023). Guideline on computerised systems and electronic data in clinical trials. https://www.ema.europa.eu/en/documents/regulatory-procedural-guideline/guideline-computerised-systems-and-electronic-data-clinical-trials_en.pdf.

[55] Fanaroff AC, Califf RM, Lopes RD (2020). New approaches to conducting randomized controlled trials. J Am Coll Cardiol.

[56] (2021). Regulation (EU) 2021/2282 of the European Parliament and of the Council of 15 December 2021 on health technology assessment and amending Directive 2011/24/EU. https://eur-lex.europa.eu/eli/reg/2021/2282/oj/eng.

[57] Huang GD, Bull J, Johnston McKee K (2018). Clinical trials recruitment planning: a proposed framework from the Clinical Trials Transformation Initiative. Contemp Clin Trials.

[58] Izmailova ES, Wagner JA, Perakslis ED (2018). Wearable devices in clinical trials: hype and hypothesis. Clin Pharmacol Ther.

[59] Rosa C, Campbell ANC, Miele GM, Brunner M, Winstanley EL (2015). Using e-technologies in clinical trials. Contemp Clin Trials.

[60] Zhou Y, Lin R, Kuo YW, Lee JJ, Yuan Y (2021). BOIN Suite: a software platform to design and implement novel early-phase clinical trials. JCO Clin Cancer Inform.

[61] Romm KL, Skoge M, Barrett EA (2025). A mobile health intervention to support collaborative decision-making in mental health care: development and usability. JMIR Form Res.

[62] Harmon DM, Noseworthy PA, Yao X (2023). The digitization and decentralization of clinical trials. Mayo Clin Proc.

[63] Kruse CS, Karem P, Shifflett K, Vegi L, Ravi K, Brooks M (2018). Evaluating barriers to adopting telemedicine worldwide: a systematic review. J Telemed Telecare.

[64] Fons-Martinez J, Ferrer-Albero C, Diez-Domingo J (2021). Assessment of the appropriateness of the i-CONSENT guidelines recommendations for improving understanding of the informed CONSENT process in clinical studies. BMC Med Ethics.

[65] (2024). Advancing clinical and translational science through accelerating the decentralization of clinical trials. https://ncats.nih.gov/sites/default/files/2024-06/RFI-DCT-Report-for-public-website-FINAL-508.pdf.

[66] Devices (smartphones and tablets) in DCTs. https://mrctcenter.org/wp-content/uploads/2023/06/Devices.pdf.

[67] de Jong AJ, Shahid N, Zuidgeest MGP (2024). Opportunities and challenges for decentralized clinical trial approaches: European health technology assessment perspective. Value Health.

[68] Fons-Martinez J, Ferrer-Albero C, Diez-Domingo J (2022). Keys to improving the informed CONSENT process in research: highlights of the i-CONSENT project. Health Expect.

[69] Murciano-Gamborino C, Pérez-Breva L, Fons-Martínez J (2026). Delphi study from a multi-stakeholder perspective to address the key challenges of decentralised clinical trials in Europe (dataset). Zenodo.

