# Supplementary material for: Addressing the Key Challenges of Decentralized Clinical Trials in Europe: Multistakeholder Perspective Delphi Study
Source: J Med Internet Res. 2026 Jul 9;28:e80625. doi: 10.2196/80625 (PMC13349229; doi:10.2196/80625)
Supplement: Multimedia Appendix 1 [file jmir-v28-e80625-s001.docx]

***Challenge 1.*** *Decentralised clinical trials may increase the burden or risk-taking for the health care providers.*

1. By paying more attention to trial safety conditions

- Provide researchers with a safety kit for home visits.
- Liability insurance policies for clinical trials should include legal coverage for all activities performed at the patient's home.
- Institutional review of risks and burdens.
- Involve the research team in the assessment of potential increased burden, training or risk of exposure and in the proposal of mitigation strategies.
- The sponsor should establish a procedure for managing the most common risks, and this training for investigators could be included in the study initiation visit.

1. By developing and improving training and support

- Explain to the research team the need for additional training in consideration of potential benefits of DCTs.
- Change management earlier in the process. Don't wait until the last minute to inform sites about the inclusion of DCT elements into a trial when it's new to them.
- Ensure early engagement with sites (already during site feasibility stage), and communicate which DCT elements will be used. Ensure that DCT provider provides adequate helpdesk support for sites and patients. Ensure that proper training material is generated.
- Trial sites/researches need to receive specific training on the decentralised aspects of the trial and the specific rules in place.
- Use of digital tools where beneficial (and it is expected that the use of such tools in many areas will increase). Trial sites/researchers need to be trained on these tools.
- Formal but short education for involved patients has to be established.
- Sufficient well-trained support staff.
- Involve study partners (for example spouse, family member or good friend) who can help the participant when problems come up during the trial. Also create a participant manual with FAQ's.
- Create clear manuals and training videos, so that the research team can always look back at the material and only study the technologies that they need at that time.
- A formal but short education can be done in different ways. Researchers have to find with tests the best form to do it.
- Increase training for patients.
- Ensure early engagement with sites (already during site feasibility stage), and communicate which DCT elements will be used. Ensure that DCT provider provides adequate helpdesk  support for sites and patients. Ensure that proper training material is generated.
- The sponsor should establish a procedure for managing the most common risks, and this training for investigators could be included in the study initiation visits.
- Allocate human resources to support the follow-up of participants' responses.
- Support trial sites/researches with trained personnel (study nurses) for the specific additional burden of decentralised trials.
- Including in the staff a new professional profile expert in digital health and in data analysis.

1. By ensuring remote follow-up of the safety of participants

- Schedule the contact moments (phone calls/telemedicine) in advance, so that the participant and their partner know when to expect a call. They can save their questions for that contact moment, unless they are very urgent. Also, if data is not coming in, this can be discussed during these contact moments.
- If continuous monitoring were to be further developed in special files, it could be fully automated, i.e. receiving a warning that a device (e.g. measuring daily motor activity) is not being used this day, etc.
- Define in the Protocol requirements for data review and no site should be expected to review data in real time.
- Intensive programming in the data collection system is needed to identify adverse effects that require contact with the HCP or intervention. Alerts for those that require assessment or action would be sent to HCP/coordinators for review. The study coordinators cannot be expected to review all the data submitted in real-time and provide timely assessment.
- Build alerts into digital tools to reduce the amount of manual review that's required.
- Optimize technology to allow participant to indicate need for clinical consultation, and have method for triaging and organizing.
- Have a protocol for events or participant reporting to identify problems in real time and then escalate to clinical attention in organized manner.
- Access to remote investigator and site staff for unplanned safety assessments requires coordination of multiple schedules (investigator, site staff, patient). Scheduling issues with telemedicine interactions may pose a risk to the timeliness, completeness, and accuracy of participant safety event reports. Since the investigator and study team do not intend to replace the participant's usual medical care, the participant's personal HCP represents a potential resource for optimising safety information collection, mitigating the duration and severity of an adverse event, and keeping the participant's HCP apprised of changes in their health status.
- Focus on patient-reported outcomes, less on measurements.
- Regarding monitoring of the data, develop a data flow at an early stage of the trial, including roles and responsibilities (e.g. PI responsibilities versus Sponsor's Medical Monitor's responsibilities). Work with DCT vendor and data management to define thresholds for automated alerts to sites/Sponsor. Communicate and engage early with sites at an early stage regarding their role with regard to monitoring data collected via DCT elements.

1. By tailoring trial set-up to DCT elements

- Procedures should be simplified as much as possible, not collect variables that will later have no clinical interest.
- If within-home monitoring or data collection is absolutely needed, perhaps limit it to a subgroup of participants of particular interest or perhaps a random smaller sample. This would avoid safety risks to the study team by minimizing the number of participants required for direct contact.
- Establish as inclusion criteria for patients some minimum requirements related to the patient's home: communications, accessibility, environment, location, resources and means available at home (dispensing and storage of medication, waste collection, for example).
- Selection right trials for DCT, for example low intervention trials.
- Protocol needs to identify which visits could be home visits or site visits. As researchers, we need to discuss this issue with the Sponsor, previously to get the final version of protocol and, we need to explain this possibility to patients in the PIS. It's important to calculate the timings. Probably a questionnaire and sampling requires 20 minutes but, if we need to process the samples or check information in a eCRF, probably they need to hold 1 hour.
- Trial sites/researches need to be supported by qualified service providers that have experience in decentralised trials (e.g. CROs, courier service, IT...).

1. Through development and selection of more adequate and standardised technology

- Use of digital tools where beneficial (and it is expected that the use of such tools in many areas will increase). Trial sites/researchers need to be trained on these tools.
- The use of AI should be explored and used.
- Having expert societies identify devices or apps (e.g. for spirometry) that meet acceptable standards for measurement. And have those societies work with the device companies to improve the precision of the measurements. In this way, there may be fewer 'recommended' devices/apps that would be used across clinical trials in a particular therapeutic area and coordinators would not have to learn a new device/app for each clinical trial. And encourage pharma to stop using homegrown data collection programs and use other standardized data systems such as REDCap.
- Standardized platforms for data collection/entry.
- Improving/choosing adequate technology (wearables).
- Use more wearable/sensor technology to track real time continuous data.
- Also easy to use with "walk me" technology so as not to require significant training.
- Make the technologies as 'dummy proof' as possible, meaning that the majority of the tasks should be automated. Steps that can be automated should be automated, before the start of the trial, so that the research team has as little work as possible.
- Allow for systems to trigger actions to reduce delays in actioning issues (using ML or AI for example).
- We need to provide better technology solutions that can integrate with other solutions and reduce multiple logins, double data entry, etc.
- Use DCT elements from 1 DCT provider via one DCT platform, to avoid that sites and patients need to handle several login data.

1. By improving collaboration and involvement of all parties involved in trial conduct

- The investigator should be involved in the design of the study to identify risks from the outset.
- Involve the research team in the assessment of potential increased burden, training or risk of exposure and in the proposal of mitigation strategies.
- Involve sites and participants into trial design so that burden can be reviewed up front.
- Ensure early engagement with sites (already during site feasibility stage), and communicate which DCT elements will be used. Ensure that DCT provider provides adequate helpdesk support for sites and patients. Ensure that proper training material is generated.
- Ensure early engagement with sites (already during site feasibility stage), and communicate which DCT elements will be used. Ensure that DCT provider provides adequate helpdesk support for sites and patients. Ensure that proper training material is generated.
- The specific roles and responsibilities of the sponsor, investigator, and any additional parties need to be clearly defined in writing and understood prior to the start of the trial.
- Regarding monitoring of the data, develop a data flow at an early stage of the trial, including roles and responsibilities (e.g. PI responsibilities versus Sponsor's Medical Monitor's responsibilities). Work with DCT vendor and data management to define thresholds for automated alerts to sites/Sponsor. Communicate and engage early with sites at an early stage regarding their role with regard to monitoring data collected via DCT elements.
- Support trial sites/researches with trained personnel (study nurses) for the specific additional burden of decentralised trials.

1. Through the development of a risk mitigation/management plan by the sponsor

- The sponsor should establish a procedure for managing the most common risks, and this training for investigators could be included in the study initiation visits.
- Ensure that a thorough risk/benefit assessment is included in the protocol to ensure that it's assessed early and accepted by regulators - This should give confidence to sites and participants regarding the inclusion of DCT elements into a trial.
- Create a Risk mitigation/management plan which can be shared with sites to provide guidance on how to proactively manage potential or experienced issues.

1. By facilitating peer-to-peer support among participants.

- Peer to peer support may reduce fears and by that also potential visits.

1. Through automation of trial procedures

- Data quality review is important, but increasingly becoming automated.
- Create automatic reminders for the research team when no data or bad quality data is coming in for a longer period of time. So change the activity of data monitoring to a passive instead of an active task.
- Allow for systems to trigger actions to reduce delays in actioning issues (using ML or AI for example).
- Regarding monitoring of the data, develop a data flow at an early stage of the trial, including roles and responsibilities. (e.g. PI responsibilities versus Sponsor's Medical Monitor's responsibilities). Work with DCT vendor and data management to define thresholds for automated alerts to sites/Sponsor. Communicate and engage early with sites at an early stage regarding their role with regard to monitoring data collected via DCT elements.

***Challenge 2.*** *Preventing challenges with logistics and management of investigational medicinal product (IMP) and biosamples.*

1. By developing training and providing support to participants regarding use of medication and collection of biosamples.

- Provide patients' homes with the necessary resources.
- Depending on the characteristics of the drug, send home the specific dose for each visit.
- Telephone support from participants.
- Scheduling telehealth calls to demonstrate appropriate collection technique   and review by  the packing materials for specimen shipment.
- Continuous follow up in courier web page for potential delays/issues in collection / shipments.
- The use of an app can help with biosampling at home. It can contain step by step guidelines for the sample collection, and literally walk them through it with diagrams, videos etc.
- Simplify study kits for participants to easily collect, store and ship samples.
- Include an adequate training for participants.
- Ensure training is provided and then people properly understand requirements and their importance.
- Training of participants in the use of medications and handling of specimens.
- For biosampling, involvement of Home Health Nurses or Televisits with the site's study team/study nurse who can instruct the patient remotely.
- Related to the management of biosamples an adequate information and training must be provided to the patient and, if it is necessary, a health-care professional must go to the patient's home to assist it.
- Provide participants with training on IMP management.
- Regarding IMP delivery to patient's homes (if allowed by local regulations and assuming that qualified vendors are used), suggest to involve Home Health Nurses or Televisits with the sites, to have a contact with the patient regarding the confirmation of correct delivery, and giving instructions regarding IMP storage and intake.
- Contracting with overnight delivery services with commitments for facilitating such work.
- Confirm with the participant the day and time to receive a drug / send an IMPs or biosample.

1. By adapting the study protocol to the therapeutic area, participant characteristics and study procedures

- Tailor research protocol according to participants / group of participants.
- Regarding drug, it depends on the trials. Sometimes you can give kits in the face to face visits at site.
- Chose or develop IMP that do not suffer from those problems
- The investigator could conduct home study feasibility surveys with candidates.
- Don't allow lab sample collections in the home for sensitive assays
- We need to detail all the procedures of the visit and which biological markers we need to analyse after sampling. Following the lab manuals we can assess the risks about the conditions accepted. This home visits modality is not available for all clinical trials and for all visits. If we need a refrigerated centrifuge immediately after the blood sampling, it could be difficult to perform at home (a refrigerated centrifuge is minimum 20kg and it's calibrated to stay in the same place, difficult to apply if we use the same centrifuge to all visits for all patients) It’s not the same if we need to process immediately and to keep at -80ºC. At the end, we need to check every detail.
- About management of biosamples: Favouring self-sampling only for well-known/routine practice devices (urine samples)
- When challenges with logistics and management of investigational medicinal product (IMP) and biosamples are foreseen, consider the possibility of integrating the DCT (a hybrid form, combining home based, traditional onsite visits, and study protocols)

1. By facilitating IMP management and temperature control

- Use automated temperature sensors for temperature-sensitive IMPs to make it obvious when temperature deviations have occurred.
- Use of an electronic device that records and stores data on when the IMP is opened and its temperature.
- Give simple instructions to the patient via a mobile phone app.
- Offer online tracking of IMP shipments to patients.
- Use smart packages with temperature control.
- Use appropriate technology/devices to track transport, delivery and administration of IMP.
- Transport the IMP or biosamples in boxes which measure the temperature/light/etc. continuously, and also upload this to a server, so that the researcher can check remotely if the correct conditions were met.

1. By facilitating BioSample management tracking

- Use QR codes to track the sample and a way for all parties to see this information.
- Transport the IMP or biosamples in boxes which measure the temperature/light/etc. continuously, and also upload this to a server, so that the researcher can check remotely if the correct conditions were met.

1. Training of professionals for the new roles and delegated tasks of the DCT

- The sponsor should have professionals trained in the delegated tasks.
- Train the professionals in charge of obtaining, handling and manipulation of biological samples in good clinical laboratory practice standards and IATA.
- Ensure training is provided and then people properly understand requirements and their importance.

1. Using local pharmacies, pick-up points, laboratories and healthcare centres

- Delivery of medication to the pharmacy office near the patient's home.
- Whenever possible, favour an easier environment for IMP management, i.e. the participant's local pharmacy, which mimics standard practice for outpatient treatment.
- Rethinking distributed delivery to focus on multiple small centres, rather than homes.
- Use of local labs, with shorter travel distance.
- Create a few distribution centres in the country in which the conditions can be controlled, where the participant can pick up the IMP or drop-off the biosamples. Pharmacies can be asked to act as a distribution centre.
- Include the home hospitalization professionals of the centres within the research team.   Have advice from home hospitalization professionals in the design of study procedures.
- To design a logistic system to collect all samples. For example to collect the material directly at home or to identify a system of point distributed on territory (lockers).

1. Use of validated products and services

- Only work with qualified service providers that have the required experience to manage such shipping.
- Follow marketed products for home-based diagnostics for collection, storage and preservation.
- Use appropriate devices/technology for sample collection.
- Use certified home biological sampling kits (e.g. from the FDA) as they can be reliably used by participants themselves.

***Challenge 3.*** *Ensuring effective collaboration with local resources.*

1. By reducing administrative burdens

- Pre-trial certification that does not need to be renewed or revisited with every new trial.
- Use vendors using site networks, and pre-established contracts with local resources.
- International quality standards.
- Inter-operability of data.
- Automate most processes, e.g. add sensors that measure temperature/light/etc. which inform the researchers when something's wrong, so that pharmacies or health centres don't need to check this.

1. By providing better training and financial resources for local healthcare professionals

- By encouraging the participation of local centres in clinical research, training their professionals in GCPs and including these professionals as members of the team.
- Financially compensate or provide more resources to centres as compensation for participation. These resources can be personnel or infrastructure.
- Implement a quality management system in these centres for trial processes and improve customer perception. This procedure could help the centre to attract other research or other clinical trials in the future.
- If these resources are performing protocol specific activities, then they will require training and closer investigator oversight. In this situation the roles should be compensated.
- Local resources should be trained and also financially compensated as well as allow them to participate in publications.
- Financial support to increase participation.
- Supporting personnel for local resources.
- Resourcing and training for local teams.
- Possibility for on-the-job training combined with easily accessible information.
- Risk-based training and risk-based implementation of legal requirements.

1. By providing better incentives and compensation for involvement of local resources in trials

- Refer participants to local care site when trial is completed.
- Promote special research centre identification for participating centres, e.g., with identification plaques on building facades that will result in a better perception by the public.
- Provide visibility in the system for centres that become certified as local research support centres.
- Inclusion in research group.
- Provide an incentive for pharmacies to participate. There would possibly be better participation from independent pharmacies vs. chain pharmacies. Chain pharmacies are focused on the financial numbers and pharmacists are unlikely to be motivated to provide this sort of service, whereas an independent community pharmacy may be interested in expanding its patient reach through participation as a research site. Pharmacies that are already specialty pharmacies in an area could be interested. The benefits to the specific pharmacy would need to be explored.
- Opportunities for career progression and inclusion in research.

1. By describing clearly the roles and functions of local partners

- Elaborate a policy for collaboration drafted through a participatory methodology with stakeholders from local settings/local resources.
- Involve members / executives from local resources to evaluate the feasibility of the proposed collaboration, and identify barriers /challenges for responsibilities and the maintenance of quality standards.
- In case no vendors are used for local resources, ensure that contracts are defined appropriately regarding roles and responsibilities. As additional contracts are required, ensure that additional workload is connected with that, so the sponsor's study team should be sufficiently resourced.
- The participation and responsibilities of the different local resources must be clearly defined and accepted/signed by the person in charge of the local resource used.
- It requires agreement by all actors. It should be specified what kind of tasks can be performed in these sites, while respecting regulations and patient confidentiality.
- Clear partnerships rather than central command and control.
- Clear description of whole process.
- Clear description of responsibilities.
- Contract between local resources and DCT centres.
- Specifications included in the contracts.

1. Making local health care providers (HCPs) and patients aware of the importance of research for patients

- The value of research to the participant and wide society should be reinforced. Many HCPs aren't looking for financial benefits but are looking for benefits to their patients.
- Reinforce the positive impact on health care of this type of study.
- Communicating clearly the benefits to them and to the patients. While there is some increased burden in some ways, there is decreased burden in other ways.   DCTs can reach more patients, in more locations, more diversity, DCTs can help reduce drop outs and increase data collection etc.

**Challenge 4.** Lack of harmonisation of the regulation and legislation.

1. By developing guidelines and facilitate/stimulate? knowledge sharing among stakeholders in DCTs

- Propose an harmonised guidance, based on interdisciplinary research and stakeholder involvement.
- Get regulatory intelligence from DCT vendor at an early stage of the trial, in order to plan accordingly on a per-country basis. Avoid building internal sponsor libraries, as regulations are fluid and are changing often. Rely on regulatory intelligence from DCT vendor.
- Elaborating guidelines like the recent guideline published by the EMA. More relation between data protection lawmakers and clinical trials lawmakers is needed to provide a common law.
- As an industry working in new innovation areas, we should aim to share our learnings, which should include highlighting the benefits of the European guidances.
- The sponsor should take every opportunity to comment on the planned regulatory guidelines and to participate in regulatory workshops.
- Development of common framework. Foster the establishment of a multi-stakeholder neutral platform to enable discussion.
- Multistakeholders approach (pharma, regulatory authority, public health and patients associations) to design the new procedures and regulatory frameworks in order to share a different use of DCTs.

1. Through gradual implementation of DCT elements incorporating adaptations to the local or national specificities.

- In the absence of homogeneity, limit the geographical scope by adapting protocols to national specificities as far as possible.
- The first step is to showcase effective DCT elements in local ECs, and drive local changes first.
- If there absence of homogeneity, local adaptations of the research protocol should be possible, so that the protocol can be adapted for cultural/local differences.
- Carry out feasibility studies in order to identify the opportunities and the challenges from a regulatory standpoint and to favour the authorization and implementation of DCTs.

1. Stimulating learning and harmonisation between EU member states/ internationally

- A common EU approach sets a standard for everyone else to follow. Early release of a guidance means that others can more efficiently follow. It should be clear that no regulatory body sets out to be vastly different to other competent authorities.
- Setting up according to the most rigid legislation for now and opening up for a cross border discussion towards a common ground.
- Need for clear mapping of variation in standards.
- Increase meetings of those involved at an international level to reach agreements.
- Working groups from different countries can be set up to work in this area.
- Learning from other European Initiatives.
- Incentives to harmonise (as seen with GCP).

1. By centralizing clinical trial ethics review at the EU level

- A central ethical review committee and data privacy officer for all European countries would be helpful. So that the protocol needs to be submitted only once, instead of in each country separately. Although we should adhere to the same European laws, review boards interpret the laws and regulations slightly different, which might result in local adaptations of the research protocol. One review board for all European countries will save time and money for both the researchers and the review boards.

1. By using advanced and verifiable digital security

- Since we want to protect the integrity of patients, patients should have more say in the level of protection.
- Improve recording of all data anonymously.
- The use of advanced and verifiable digital security is essential to allow for e-signatures and protected data collection.

1. Through specialisation in DCT roles.

- Regarding the current variety and, in consequence, increased operational complexity: ensure  to have a dedicated function in the study team for DCT element implementation

***Challenge 5.*** *Improving on the lack of specific knowledge and accumulated experience for ethical, legal and regulatory assessment of DCTs.*

1. Through general knowledge-sharing, education and training for conducting DCTs

- Organise sessions or conferences inviting professionals of different profiles to give their vision and talk about their experience in participating in these studies.
- Organise practical workshops on the management and development of the different stages.  Carry out practical exercises on the development of some key visits such as the inclusion of the patient with the obtaining of the IC, a follow-up visit at home with delivery of medication and biological samples... these exercises can be carried out with the help of an illustrative video.
- Promoting courses on good clinical practice in these studies.
- Promote national working groups that include professionals with these profiles to be disseminated with the help of industry and regulatory agencies.
- Promote scientific and ethical publications and research.
- Proactive sharing of lesson's learned via industry stakeholder meetings such as DIA, CTTI, local forums, etc., as well as regulator led forums such as EMA stakeholder meetings.
- Hold regular webinars/experience sharing for any stakeholders.
- Have forums at regional and national level where dialogue can take place with several Ethics Committees at the same time. This would make it easier to initiate information exchange sessions.
- Trials@Home is building a great reputation so expanding on that, potentially by attending more conferences, publishing white papers etc., creates a lot of awareness.
- Facilitate training.
- Working groups can be set up to share experience and knowledge.
- Organise dissemination seminars and training courses.
- Join cross-company initiatives. Use and promote tools developed by cross-company alliances and initiatives. E.g.  MCTC ('Modernising Clinical Trial Conduct') initiative by Transcelerate, which is providing tools also promoting DCT elements at congresses.
- Making training and formation (free online courses for example).
- Organising dedicated multi-disciplinary workshops and congresses & webinars.
- Organise symposiums on DCTs that provide sufficient time for discussion, such as the one held during the PRIM&R (Public Responsibility in Medicine and Research) conference in December 2022. This conference is aimed at Institutional Review Board personnel, who can comment on ethical considerations.
- There are other annual conferences focused on clinical trial design and execution, so repeating the message will be important as more investigators gain experience with DCT who can share and comment on the issues cited above.
- Availability of training delivered by public sector partners.
- Publication and webinars of experiences.
- Arrange training via CTIS or via EMA.
- Connect to studies with other decentralised elements, also learning from studies during COVID.

1. By promoting harmonisation of guidelines at European level and continuous dialogue with regulatory agencies

- Through research for adequate assessment tools and review process.
- Elaborating guidelines like the DCT EMA guideline, including the different stakeholders involved.
- Justifying why is feasible to offer some visits at home in a specific trial. Probably, we can design a check list, asking about the home conditions required, human resources needed, equipments and materials,...
- Guidelines and documents endorsed at international/European level.
- Set up working groups to involve the ECs at every step- not just showcasing results but involve them early in the conversation so they can have input on the design of the studies. Try out many small pilot studies to test various elements first.
- Clear agreed standards of best practice.
- Ask for scientific advise at (centralised) regulatory authorities.
- Communicate with inspectorate institutes.
- Write guidelines for the different stakeholders, which are all in line with each other.
- Use multistakeholders approach (pharma, regulatory authority, public health and patients associations) to design the new procedures and regulatory frameworks in order to share a different use of DCTs.

1. By building expertise on DCTs and move towards centralised decision making.

- More centralised Ethics Boards.
- Centralise use of experts through video visits.
- Have experts oversee the team and technicians, and use triage for escalation of issues that require expertise.
- Professionalise participation in Committees.
- To provide a knowledge base for Ethics Committees.

1. By simplifying and optimizing technology to reduce complexity.

- Simplify and optimise technology for all parties to reduce complexity

***Challenge 6.*** *Overcoming barriers due to the use of digital technologies.*

1. By developing and improving training and support for participants and caregivers

- Unless absolutely necessary and justified in the trial protocol, participants should not be excluded from trials due to technology reasons. It should be down to sponsors to offer support for potential challenges, as far as is reasonable.
- User training can be facilitated and logistical support can be improved in the event of incidents. It is common that if the application stops working one day the patient does not use it again, with the great loss of data that this entails.
- Online help (hotline) possibility for participants to contact trial staff in case of difficulties
- Ensure proper helpdesk support (in local language!) and training of patients.
- We have to use telehealth visits to ensure correct use of the device during data collection – at least until quality is ensured. Having excellent tech support is essential – the coordinators can be taught to do some troubleshooting but it could become burdensome so the manufacturer needs to be committed to providing tech support.
- Training participants in the use of digital tools.
- Initial setup visits with participants.
- Have video explanations available for participants to consult.
- Always offer Home Health Nursing support for patients to handle DCT devices.
- Training and education courses for carers, patients and caregivers.
- Digital tutor to help the patients and caregiver to face the digital procedures.

1. By making sure sufficient financial and technological resources are available to participants.

- Unless absolutely necessary and justified in the trial protocol, participants should not be excluded from trials due to technology reasons. It should be down to sponsors to offer support for potential challenges, as far as is reasonable.
- Provide hotspots for people who don't have wifi (although that doesn't solve the problem of poor mobile phone service in remote areas).
- Providing devices (tablets) to participants to use for telehealth and data entry.
- Making them sign a contract before giving them an expensive electronic device stating that they will take good care of the device while participating in the study.
- Helping participants delete study applications from the device after the study is over.
- Compensation to participants (electricity e.g. if we need to connect the centrifuge or other equipment) or for using their laptop.
- Provide devices to participants so that they do not have to own one.
- Encourage staggered recruitment of patients when the use of the devices is essential, providing the centre with a certain number of devices that have to be reused by several patients.
- If trial participants are baring the costs of technology, these must be reimbursed. We should also pay upfront and not expect participants to be out of pocket.
- Sponsor must provide all the devices to patients. Any expense must be reimbursed.
- To prepare a budget considering all extra human and materials, taking into account that we have less costs related to the use of hospital facilities.
- Decrease costs: let participants bring their own device. Ask developers to provide their devices/apps for free and in return give them the (anonymous) data.
- Provision of local community internet hubs/signal boosts associated with the trial (and that remain afterwards).
- Specific assurance in order to guarantee the digital protection of people.
- To decrease the economic burden for patients and caregivers on digital health (internet, device, assurance, protection procedure, privacy and digital security).
- To decrease the digital divide in particular on privacy and cybersecurity for people.

1. By making on-site/offline alternatives to decentralised elements available.

- Don’t force participants to DCT. The participant should always have the choice to perform assessments also on-site, depending on the patient's preference. The individual patient needs should be in the focus. There is no 'one size fits all'.
- If the problem is the electronic connection, we can use paper as source document in some places.
- If the participants prefers, we can offer the use of classic consent instead of eConsent.
- Plan the eConsent discussion always with a televisit, or offer the patient a face to face on-site discussion (depending on patient's preference).
- Let participants device how to communicate using their preferred method (e.g. video calls, phone calls, visits).

1. By simplifying and adapting technology for participants’ ease of use

- Use of older, more available technology.
- Make sure devices work without internet. For example save data offline and upload once internet connection is established.
- Make the study procedures easy to understand and provide a manual.
- Assess case by case specific challenges related to vulnerable groups and the use of technologies, including in advance the assess of potential digital divide, etc.
- Encourage the use of patient devices and favour the design of open source or free format programs. Guarantee security measures in these cases.
- Feedback to tech companies/OS developers to maintain backwards compatibility.

1. By ensuring data quality of remote/digital technologies used in DCTs

- Optimize web portals for data collection.
- Use devices with CE mark.
- Data Protection Impact Assessment is needed.
- Real-time data monitoring so that researcher can intervene in time. Start data analyses early (after couple of participants are finished), so that possible mistakes can be identified early in the data collection process. Make sure that the researchers and participants have to do as little as possible, i.e., automate all processes as much as possible to prevent human errors.
- Improved technology to identify device failure.
- Make the measurement time as long as possible to reduce signal/noise ratio.

1. Through local resources involvement.

- Seek the support of local centres for these procedures.
- Provision of local community internet hubs/signal boosts associated with the trial (and that remain afterwards).
- Hospitals must provide adequate spaces and a good connectivity.

1. By centralising the DCT elements used in a single vendor

- Use only 1 DCT vendor for all DCT elements, to avoid patients struggling with several systems/log in data.

1. By ensuring that discussion between the researcher and the potential participant is maintained as part of the informed consent process.

- It is an important aid to obtain informed consent, always understanding that it is an aid.  A member of the research team must always be present to answer questions.
- Electronic consents that are simply digital forms of paper documents are insufficient in and of themselves for adequate informed consent. Ensure that a face to face conversation (includes just telephone but telehealth is now preferred) occurs prior to signing consents. This ensures that the participant fully understands the commitment and reduces lost to follow-up.
- Provide the participant with the signed forms either digitally or hard copy mailed to them.
- Virtual real time-face to face discussion, common informative sessions.
- Be very transparent about our DCTs to participants and proactively discuss expectation and potential challenges. The more participants understand what is expected from the outset, the less impact issues that do arise will have.
- All the information must be included in the information sheet signed remotely or using the print-to-sign method.
